# Pancreatic cancer grading in pathological images using deep learning convolutional neural networks

**DOI:** 10.12688/f1000research.73161.2

**Published:** 2022-11-01

**Authors:** Muhammad Nurmahir Mohamad Sehmi, Mohammad Faizal Ahmad Fauzi, Wan Siti Halimatul Munirah Wan Ahmad, Elaine Wan Ling Chan

**Affiliations:** 1 Faculty of Engineering, Multimedia University, Cyberjaya, Selangor, 63100, Malaysia; 2 International Medical University, Kuala Lumpur, Kuala Lumpur, Malaysia

**Keywords:** digital pathology, pancreatic cancer, cancer grading, deep learning, image classification

## Abstract

**Background:** Pancreatic cancer is one of the deadliest forms of cancer. The cancer grades define how aggressively the cancer will spread and give indication for doctors to make proper prognosis and treatment. The current method of pancreatic cancer grading, by means of manual examination of the cancerous tissue following a biopsy, is time consuming and often results in misdiagnosis and thus incorrect treatment. This paper presents an automated grading system for pancreatic cancer from pathology images developed by comparing deep learning models on two different pathological stains.

**Methods:** A transfer-learning technique was adopted by testing the method on 14 different ImageNet pre-trained models. The models were fine-tuned to be trained with our dataset.

**Results:** From the experiment, DenseNet models appeared to be the best at classifying the validation set with up to 95.61% accuracy in grading pancreatic cancer despite the small sample set.

**Conclusions:** To the best of our knowledge, this is the first work in grading pancreatic cancer based on pathology images. Previous works have either focused only on detection (benign or malignant), or on radiology images (computerized tomography [CT], magnetic resonance imaging [MRI] etc.). The proposed system can be very useful to pathologists in facilitating an automated or semi-automated cancer grading system, which can address the problems found in manual grading.

## Introduction

Pancreatic cancer is one of the most lethal malignant neoplasms in the world, ^1^ developed when cells multiply and grow out of control in the pancreas, ^2^ forming cancer cells caused by cells mutation in their genes. ^3^ Doctors commonly perform a biopsy to diagnose cancer when physical examination or imaging tests like magnetic resonance imaging (MRI) and computerized tomography (CT) scans are insufficient. In pancreatic cancer, grading is essential for planning treatment but is currently done using a meticulous microscopic examination. ^4^ Limited work found on analysis of pathological images for pancreatic cancer. Niazi *et al.* ^5^ presented a deep learning method to differentiate between pancreatic neuroendocrine tumor and non-tumor regions based on Ki67 stained biopsies. The purpose is for the quantification of positive tumor cells in a hotspot. Up to now, there has been no successful implementation of artificial intelligence (AI) for classifying pancreatic cancer grade. The absence of such AI work motivates this paper to use transfer-learning to grade pathological pancreatic cancer images using 14 deep learning (DL) models. This work can facilitate an automated cancer grading system to address the exhaustive work of manual grading.

### Contributions

This work presents an automated grading system focusing on pancreatic cancer from pathology images, which has not been done before to the best of our knowledge. The work also contributes a comparison of performance for 14 DL models on two different pathological stains, namely the May-Grünwald-Giemsa and haematoxylin and eosin.

### Pancreatic cancer and digital pathology

Pancreatic cancer is considered to be under-studied, and improvements in the diagnosis and prognosis of pancreatic cancer have therefore been minor. ^6^ Digital pathology is an image-based environment obtained by scanning tissue samples from glass slides. Staining, usually using May-Grünwald-Giemsa (MGG) and haematoxylin and eosin (H&E) stains, is carried out on the tissue samples before digitization into whole-slide images. The cancer grade is identified by the degree of differentiation of the tumour cells ^7^ ranging from well to poorly differentiated as described in Table 1.

**Table 1.** Pancreatic cancer grade. MGG = May-Grünwald-Giemsa; H&E = haematoxylin and eosin.

Grade	Normal	Grade I	Grade II	Grade III
**Description**	Benign. Cells are not cancerous and will not spread.	Well differentiated. Cancer cells look like normal cell and are not growing rapidly.	Moderately differentiated. Cancer cells look abnormal and are growing faster than normal cell.	Poorly differentiated. Cancer cells look very abnormal and may spread aggressively.
**MGG Stain**	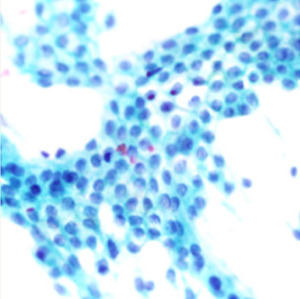	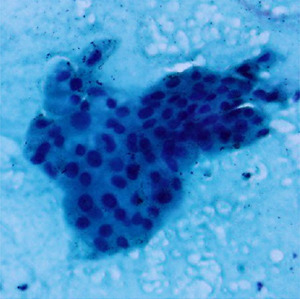	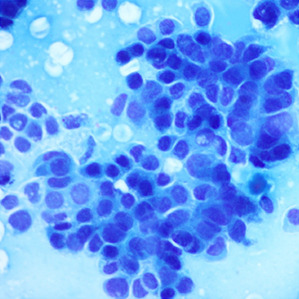	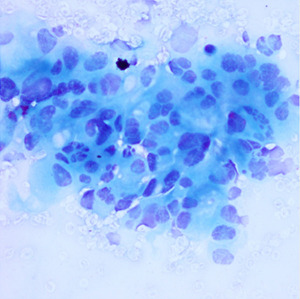
**H&E Stain**	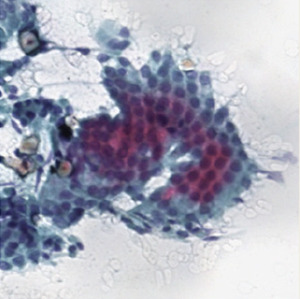	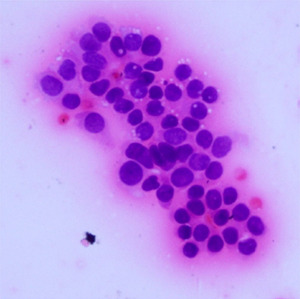	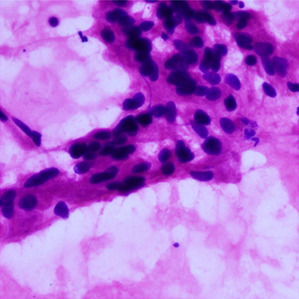	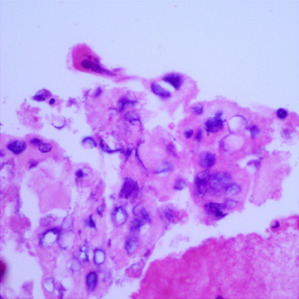

### Deep learning and related works

Convolutional neural network (CNN) is a widely used deep learning (DL) algorithm in medical image-based classification and prediction. ^8^ Several methods use CNN in cancer detection and diagnosis ^9^ such as the Gleason grading of prostate cancer, ^10^^–^^12^ colon cancer grading, ^13^ breast cancer detection, ^14^^,^^15^ and pancreatic cancer detection ^16^^–^^19^ and classification. ^20^ AI has been proven to assist clinicians with better prediction and faster diagnosis for breast cancer screening. ^21^ However, grading of pancreatic cancer with DL still needs comprehensive study.

## Methodology

**Figure 1.**
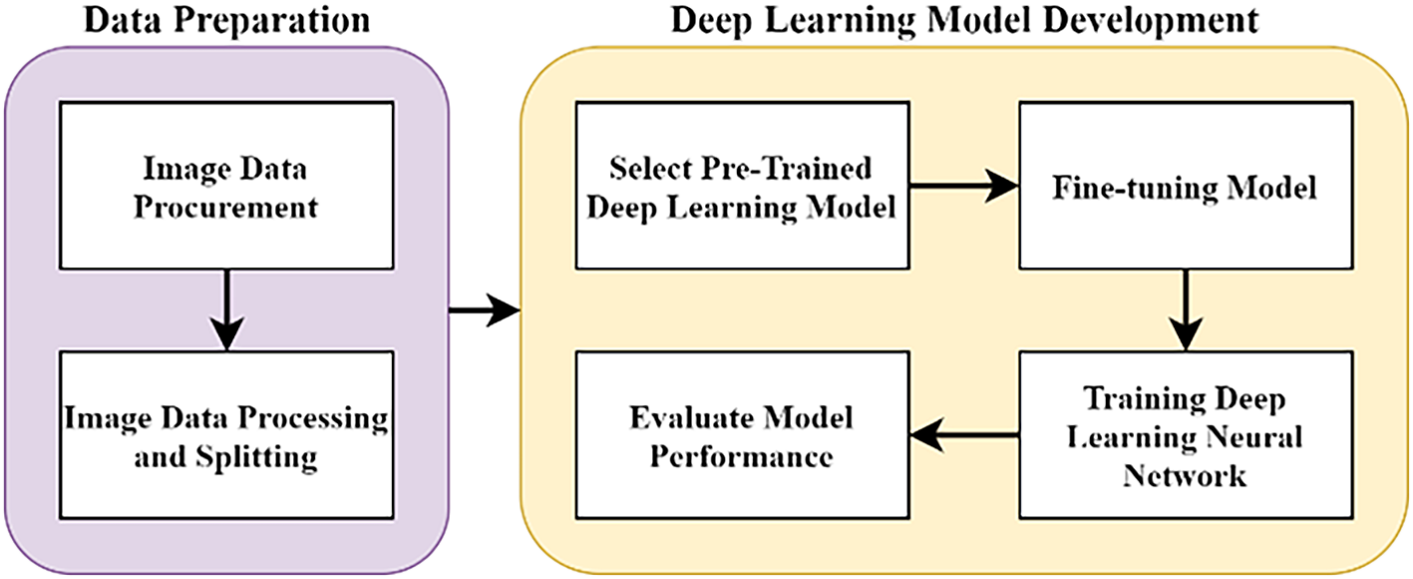
Flowchart of the research work.

The methodology of this work was done at Multimedia University, Cyberjaya, from June 2020 to May 2021. The overall methodology of this research is as illustrated in Figure 1, with two major stages. In the data preparation stage, pathology images of pancreas tissue samples were obtained from our collaborator and the images were pre-classified by a pathologist into four classes. In the DL model development stage, the images were trained on the DL model and evaluated accordingly. All stages were carried out using Jupyter notebook in Google Colab. The source code is available from GitHub and archived with Zenodo. ^26^

### Ethical approval

This work was approved by the Research Ethics Committee of Multimedia University with approval number EA2102021. This article does not contain any studies with human participants or animals performed by any of the authors. Only pathology images were used, and the patients’ personal data were anonymized.

### Dataset preparation

**Pathology image procurement**

A total of 138 high-resolution images with varying dimensions (1600 × 1200, 1807 × 835 and 1807 × 896) were obtained and pre-classified by the collaborators (see *Acknowledgements*). Four classes were identified (as shown in Table 2): Normal, Grade-I, Grade-II and Grade-III. Each image consisted of a tissue-sample stained with MGG and H&E. The image distribution in each class was unequal with Grade-II having 58 images and Normal with only 20 images. To better capture the cells characteristics which is paramount in determining their grade and to match the lower-resolution setting of the network’s input, the images were pre-processed into small non-overlapping patches.

**Table 2.** Number of high-resolution images in the dataset. MGG = May-Grünwald-Giemsa; H&E = haematoxylin and eosin.

Stain\Class	Normal	Grade I	Grade II	Grade III	Total
MGG stained	13	4	43	19	**79**
H&E stained	7	27	15	10	**59**
**Total**	**20**	**31**	**58**	**29**	**138**

**Image pre-processing**

**Figure 2.**
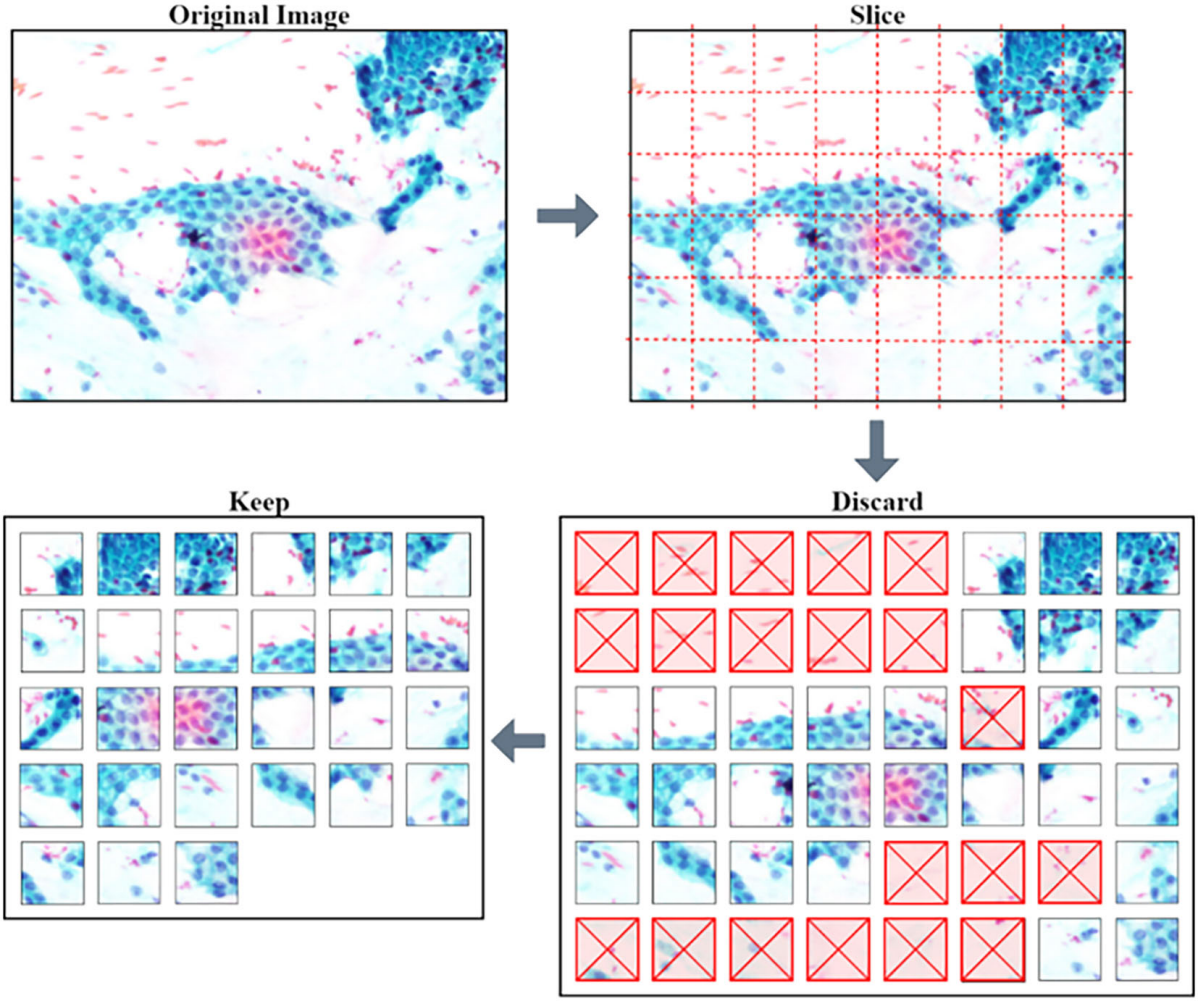
Process of slicing an image and discarding unwanted non-tissue patches.

The pre-trained models require a low dimension and square image for training and making predictions. The squared slicing method is used where smaller patches with approximately 200 × 200 pixels of non-overlapping regions are sampled from the original images. Further processing was done to remove unwanted patches, as shown in Figure 2.

**Image dataset**

A total of 6468 patches were generated from the slicing process of the 138 original images which is an increase of 468% in number of images. Overall, 50.5% (3267) of the patches with background and non-tissue information were discarded, and the remaining are listed in Table 3. Examples of MGG stain and H&E stain pathology images are shown in Table 1, with the mixed dataset combining all images from MGG and H&E stains. From the numbers in Table 3, these datasets still had an imbalanced number of patch images in each class but this can be mitigated by employing a weighted average to evaluate the model.

**Table 3.** Number of sliced images kept for training and validation. MGG = May-Grünwald-Giemsa; H&E = haematoxylin and eosin.

Stain\Class	Normal	Grade I	Grade II	Grade III	Total
MGG stained	401	108	983	366	**1858**
H&E stained	139	606	309	289	**1343**
**Total**	**540**	**714**	**1292**	**655**	**3201**

**Training-validation splitting and K-fold cross-validation**

To evaluate the DL model, images in each dataset were split into training and validation set with 80-20 ratio. K-fold cross-validation with K = 5 was used by splitting all MGG, H&E and the mixed dataset into five parts, producing cross-validation sets with five new copies of MGG, H&E and mixed datasets and labelled (e.g. MGG Set 1 to MGG Set 5 for MGG). Each set had a different set of images used for training (80%) and validation (20%). The average value was calculated from the five-training iterations to evaluate the performance.

**Image data augmentation and normalisation**

Image data augmentation was implemented to virtually expand the training set, but not on the validation set. The transformation parameter involved was horizontal flip, vertical flip and -90° to 90° rotation range. Image data normalisation was used to rescale image pixels from a range of [0,255] to [0,1] so the input pixels will have similar data distribution.

### CNN deep learning model development

Deep CNN algorithm was used for developing a model for classifying pancreatic cancer grading from pathology images.

**Transfer-learning**

A total of 14 CNN pre-trained models from recognizing 1000 classes in ImageNet was selected from Keras API ^22^ to get the best model for classifying the 4-grade classes of pancreatic cancer. The proposed pre-trained models are listed in Table 4 along with their original model’s image input shape, and its respective top-1 accuracy on the ImageNet validation set.

**Table 4.** ImageNet pre-trained models.

Pre-trained models	Input shape	Top-1 accuracy
Xception	299 × 299	0.790
VGG16	224 × 224	0.713
VGG19	224 × 224	0.713
ResNet50V2	224 × 224	0.760
ResNet101V2	224 × 224	0.772
ResNet152V2	224 × 224	0.780
InceptionV3	299 × 299	0.779
InceptionResNetV2	299 × 299	0.803
MobileNetV2	224 × 224	0.713
DenseNet121	224 × 224	0.750
DenseNet169	224 × 224	0.762
DenseNet201	224 × 224	0.773
NASNetMobile	224 × 224	0.744
NASNetLarge	331 × 331	0.825

**Fine-tuning**

All 14 models were fine-tuned with four newly added layers to extract the features from pathology images: a flatten-layer to form a 1D fully connected layer; a dense-layer with 256 nodes and ReLu activation-function; a dropout-layer with a rate of 0.4 to regularise the network; and lastly another dense-layer with 4-nodes and SoftMax activation-function to normalize the probability of prediction.

**Setup and evaluation parameters**

Batch size of 64 was chosen to allow the computer to train and validate 64-patch-samples at the same time. Adam optimizer with default initial learning rate of α = 0.01 and moment decay rate of β1 = 0.9 and β2 = 0.999 was used. The loss function is calculated using categorical cross-entropy for the 4-class classification task. With this setup, the models are compiled and trained for 100-epochs.

The confusion matrix, precision, recall, f1-score and weighted-average were used to evaluate the model's performance. Weighted-average was used to calculate the performance of individual cross-validation set and suitable for imbalanced dataset. The equation for the weighted-average is:

$$\text{averag}e_{\left(\text{weighted}\right)}=\sum_{k=1}^{n}\left(P_{k}\frac{\text{No. of images in class}\;k}{\text{Total number of images in dataset}}\right)$$

## Results and discussion

### Effect of data augmentation

This experiment was done with the first cross-validation set of the mixed dataset, to observe how data augmentation impacts a model training performance. ^25^ Table 5 and Table 6 display the final accuracy and loss of training and validation set after 100 epochs. Without data augmentation in Table 5, it is evident that overfitting has occurred, because the model is doing very well on the training set but not on the validation set. With data augmentation, the validation accuracy improved, specifically on VGG19 model (54.83% to 77.22%). The training accuracy of other models are slightly reduced with data augmentation (except for VGG19) but it is normal as the model is learning newly transformed images. The validation loss also shows a reduction, as in Table 6, such as on NASNetLarge model from 3.36376 to 0.68587. Overall, these results show that data augmentation may reduce overfitting and improve model performance as reported in. ^11^^,^^14^^,^^15^ The reason behind this is the model is becoming more robust with data augmentation for getting to learn various transformed images of the limited size dataset. These kind of images are high-likely to exist in real-world applications, especially when it comes to unique human cells.

**Table 5.** Model accuracy for without and with data augmentation after 100 epochs.

Pre-trained models	Without image data augmentation		With image data augmentation	
	Training set (%)	Validation set (%)	Training set (%)	Validation set (%)
Xception	99.88	83.75	93.63	85.02
VGG16	97.35	76.88	86.48	81.12
VGG19	64.85	54.83	77.34	77.22
ResNet50V2	100.00	82.66	95.39	86.11
ResNet101V2	99.92	79.84	93.44	85.80
ResNet152V2	100.00	81.25	95.35	86.58
InceptionV3	99.38	82.65	91.23	83.15
InceptionRes-NetV2	99.61	79.06	90.12	83.78
MobileNetV2	99.68	82.50	94.14	85.02
DenseNet121	99.84	82.81	94.69	88.14
DenseNet169	99.84	85.00	95.98	89.70
DenseNet201	99.92	85.47	96.76	88.14
NASNetMobile	99.92	79.69	91.21	81.75
NASNetLarge	98.32	73.48	89.84	79.88

**Table 6.** Model loss for without and with data augmentation after 100 epochs.

Pre-trained models	Without image data augmentation		With image data augmentation	
	Training set	Validation set	Training set	Validation set
Xception	0.00241	1.49118	0.16254	0.47146
VGG16	0.09256	0.87281	0.35639	0.46890
VGG19	0.95239	1.14815	0.56213	0.60279
ResNet50V2	0.00005	1.40010	0.12776	0.42690
ResNet101V2	0.00287	1.31044	0.17701	0.41832
ResNet152V2	0.00010	1.68771	0.12355	0.40160
InceptionV3	0.01608	1.02646	0.23775	0.44671
InceptionRes-NetV2	0.02074	1.15162	0.26173	0.47013
MobileNetV2	0.00703	1.03879	0.14739	0.47216
DenseNet121	0.00615	0.92605	0.14379	0.33270
DenseNet169	0.00423	0.93888	0.11209	0.35975
DenseNet201	0.00385	0.94979	0.09399	0.32288
NASNetMobile	0.00338	1.80272	0.22978	0.47335
NASNetLarge	0.05669	3.36376	0.32225	0.68587

### Comparison analysis of model performance

**Figure 3.**
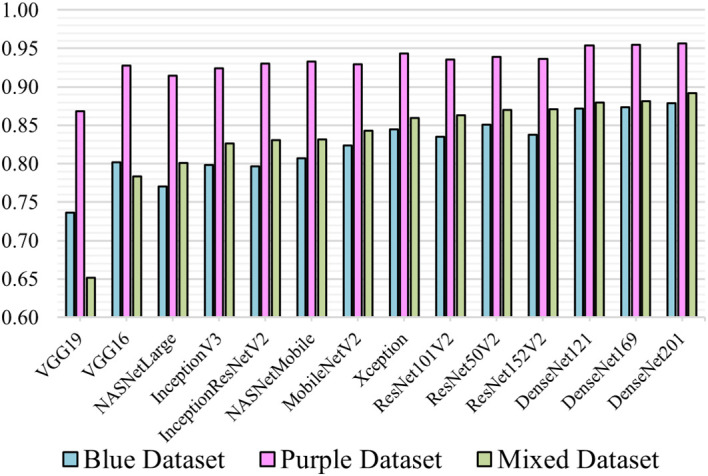
Mean F1-score of models.

The overall performance results of all 14 different transfer-learning models proposed for this experiment are presented. Each model was trained with the 3 datasets and 5-fold cross-validation. Figure 3 illustrates the overall performance in terms of mean f1-score.

**Comparison between MGG, H&E and the mixed dataset**

This comparison shows how a DL model learns from single coloured stain. In Figure 3, all models trained with the H&E obtained the highest f1-score compared to MGG and mixed. Most models scored above 0.9 except for VGG19 (0.87). When trained with the MGG, models other than VGG16 and VGG19 performed the lowest compared to H&E and Mixed. The performance of mixed is as expected because it contains a mixture of both datasets. The VGG16 and VGG19 model, however, performed better on MGG than Mixed, due to the small VGG network architecture and small fully-connected layers making it unable to learn complex features and patterns in pathology image. The trend described in Figure 3 indicates that image patches in the H&E are easier to learn with better prediction than MGG.

**Comparison between pre-trained models**

From the result, DenseNet network architecture was the best at classifying pathology images where all three variations trained on MGG, H&E and mixed take the top spot among the 14 models. The ResNet on mixed dataset were ranked ascendingly from ResNet101V2, ResNet50V2 and ResNet152V2 before the three DenseNet models. This supports the work of Huang *et al.* in ^23^ where DenseNet was designed to improve the ResNet architecture. DenseNet201 which is a much deeper layer than the other two DenseNet models managed to achieve the highest f1-score of 0.88, 0.96 and 0.89 for MGG, H&E and mixed, respectively. The DenseNet121 and DenseNet169 performance on the three dataset scores were marginally lower at 0.87, 0.95, 0.89 and 0.87, 0.95, 0.88, respectively. This shows that a deeper DenseNet layer can perform more accurate prediction.

The Xception ^23^ and InceptionResNetV2 ^24^ are improvements of InceptionV3, which performs better than their ancestor. The f1-scores for Xception trained by MGG, H&E and Mixed are 0.85, 0.94 and 0.86, as compared to InceptionV3, 0.80, 0.92 and 0.83, respectively. However, InceptionResNetV2 are just slightly higher than InceptionV3 (0.93 and 0.83 for H&E and mixed) but lower for MGG (0.80). VGG models did not perform quite well when compared to its earlier models. VGG19, which is supposed to be an improvement to VGG16, failed to achieve a greater f1-score, with 0.74, 0.87 and 0.65 for the MGG, H&E and mixed, respectively, while VGG16 was higher at 0.80, 0.93 and 0.78. The results concluded that VGG19 was the worst performing model for our datasets.

This experiment applied transfer-learning on 14 ImageNet pre-trained models to classify pancreatic cancer grades. From the comparisons, DenseNet201 model is suggested for practical application of pancreatic grading system of MGG or H&E stains.

**Comparison between the best and the worst performing model**

Table 7 and Table 8 shows the precision and recall of VGG19 (the worst) and DenseNet201 (the best) for the three datasets. VGG19 struggles to make prediction for Grade-I patches in MGG where the precision and recall are 0.00 for CV sets 3, 4, and 5. A similar pattern is noticed in Grade-III patches, and from our observation, this is because most of the Grade-I and Grade-III patches were wrongly predicted as Grade-II. This is due to the imbalance classes in MGG where Grade-II patches account for 52.9% of the total images whereas Grade-I consist of only 5% and Grade-III 19.7%. This class imbalance has caused the VGG19 model to struggle a lot at recalling class with fewer data.

**Table 7.** Precision rate of VGG19 and DenseNet201.

Precision		VGG19					DenseNet201				
Class\CV set		1	2	3	4	5	1	2	3	4	5
**MGG Dataset**	Normal	0.87	0.91	0.96	0.89	0.85	0.95	0.93	0.99	0.94	0.95
	Grade I	0.62	0.64	0.00	0.00	0.00	0.77	0.85	1.00	0.79	0.70
	Grade II	0.77	0.80	0.74	0.72	0.77	0.87	0.87	0.91	0.86	0.90
	Grade III	0.71	0.56	0.54	0.56	0.64	0.79	0.83	0.80	0.85	0.84
	Weighted Average	0.77	0.77	0.70	0.68	0.72	0.87	0.87	0.91	0.87	0.89
	**Mean**	**0.7289**					**0.8819**				
**H&E Dataset**	Normal	1.00	0.96	0.96	1.00	0.96	1.00	1.00	1.00	1.00	1.00
	Grade I	0.98	0.95	0.97	0.91	0.91	0.98	1.00	0.99	0.97	0.97
	Grade II	0.92	0.89	0.84	0.90	0.86	0.93	0.94	0.87	0.95	0.86
	Grade III	0.95	0.75	0.81	0.90	0.93	0.92	0.93	0.88	0.98	0.95
	Weighted Average	0.89	0.87	0.89	0.88	0.88	0.96	0.97	0.94	0.97	0.94
	**Mean**	**0.8802**					**0.9565**				
**Mixed Dataset**	Normal	0.90	0.86	0.69	0.76	0.61	0.94	0.88	0.89	0.96	0.94
	Grade I	0.95	0.95	0.90	0.81	0.87	0.93	0.97	0.98	0.96	0.95
	Grade II	0.98	0.67	0.65	0.52	0.56	0.85	0.85	0.89	0.88	0.87
	Grade III	0.78	0.88	0.88	0.00	0.00	0.83	0.86	0.82	0.91	0.83
	Weighted Average	0.92	0.81	0.76	0.52	0.52	0.88	0.88	0.90	0.92	0.89
	**Mean**	**0.7055**					**0.8935**				

**Table 8.** Recall Rate of VGG19 and DenseNet201.

Recall		VGG19					DenseNet201				
Class\CV set		1	2	3	4	5	1	2	3	4	5
**MGG Dataset**	Normal	0.89	0.93	0.90	0.84	0.88	0.89	0.97	0.93	0.94	0.94
	Grade I	0.23	0.41	0.00	0.00	0.00	0.45	0.50	0.55	0.52	0.76
	Grade II	0.92	0.90	0.93	0.94	0.93	0.94	0.94	0.95	0.94	0.94
	Grade III	0.47	0.42	0.37	0.30	0.44	0.77	0.71	0.88	0.73	0.71
	Weighted Average	0.78	0.78	0.76	0.74	0.77	0.87	0.88	0.91	0.87	0.88
	**Mean**	**0.7669**					**0.8819**				
**H&E Dataset**	Normal	1.00	0.96	0.96	0.93	0.82	1.00	1.00	1.00	1.00	1.00
	Grade I	0.80	0.91	0.93	0.91	0.93	0.99	0.99	0.98	0.99	0.97
	Grade II	0.92	0.90	0.92	0.97	0.87	0.87	0.94	0.89	0.95	0.92
	Grade III	0.72	0.83	0.76	0.76	0.72	0.95	0.95	0.90	0.93	0.90
	Weighted Average	0.84	0.87	0.88	0.87	0.86	0.95	0.97	0.94	0.97	0.95
	**Mean**	**0.8651**					**0.9571**				
**Mixed Dataset**	Normal	0.76	0.81	0.92	0.29	0.57	0.94	0.98	0.97	0.98	0.94
	Grade I	0.75	0.78	0.74	0.79	0.78	0.90	0.87	0.89	0.92	0.85
	Grade II	0.95	0.95	0.92	0.93	0.90	0.90	0.92	0.91	0.94	0.92
	Grade III	0.46	0.37	0.11	0.00	0.00	0.78	0.75	0.82	0.81	0.82
	Weighted Average	0.77	0.77	0.71	0.60	0.63	0.88	0.88	0.90	0.92	0.89
	**Mean**	**0.6981**					**0.8933**				

For H&E images, the effect of class imbalance however did not affect the performance of VGG19. The recall and precision for Normal class are ranked among the highest despite its smallest number (10%) of patches. Looking back at Table 1, the H&E Normal images have a quite different stain colour compared to other classes, which explains the good prediction for both models. This could be seen as a problem where limited image variation can cause biasness. The precision of Normal class would score poorly if it were tested to predict different variation of H&E stain image even with the same set of ground truth, but can be assuaged if the class have many different variations of stain colour.

For the mixed dataset, VGG19 also struggled to predict Grade-III class, especially on CV 4 and 5 where it scored 0.00 for both metrics. The reason could be that the Grade-III patches are difficult for the VGG19 model to learn. This is the reason why cross-validation should be performed to rigorously evaluate a DL model. DenseNet201 managed to get good recall for Grade-III patches for both CV sets, confirming its ability to learn complex features on the pathology image.

From the study, we can see that integrating AI into the diagnosis system can assist the pathologist in getting the suggestive grading based on the prediction. It is however meant to assist and not on decision-making. The future aim of this study is to have a platform for screening of pancreatic cancer biopsies.

## Conclusion

This paper presents development of several deep learning models through transfer-learning for classifying pancreatic cancer grade from pathology images. The datasets were trained on a total of 14 ImageNet pre-trained models. Image data augmentation was performed to counter the low number of images and has proven to improve the validation accuracies of all pre-trained models up to 40%. The evaluation on 14 pre-trained models shows that the DenseNet models performed best compared to the other models. Most of the models trained by H&E managed to achieve f1-score above 0.9. The MGG dataset scores lower f1-score compared to the mixed dataset. The highest f1-scores were achieved by DenseNet201, with 0.8786, 0.9561 and 0.8915 for MGG, H&E and Mixed, respectively. To the best of our knowledge, no similar work on pancreatic cancer grading has been reported in the literature. With these promising early results, this work can aid pathologists in facilitating an automated pancreatic cancer grading system for better cancer diagnosis and prognosis. This study has not been tested with whole slide images (WSI), but similar approaches can be applied. Further improvements to the system can potentially be achieved by using future state-of-the-art DL models.

## Data availability

### Underlying data

Open Science Framework: Dataset for Pancreatic Cancer Grading in Pathological Images using Deep Learning Convolutional Neural Networks. https://doi.org/10.17605/OSF.IO/WC4U9. ^25^

This project contains the following underlying data:

- Dataset PCGIPI-Original.zip (pancreatic pathological image patches used for our analysis. The stain types are May-Grünwald-Giemsa (MGG) and Haematoxylin and Eosin (H&E)).
- Dataset PCGIPI-sliced.zip
- PCGIPI Results.xlsx
- Slicing Process for Table 3.docx

Data are available under the terms of the Creative Commons Zero “No rights reserved” data waiver (CC0 1.0 Public domain dedication).

### Extended data

Analysis code available from: https://github.com/mnmahir/FYProject-PCGIPI

Archived analysis code as at time of publication: https://doi.org/10.5281/zenodo.5532663. ^26^

License: MIT
