# Supplementary material for: Pancreatic cancer grading in pathological images using deep learning convolutional neural networks
Source: F1000Res. 2022 Nov 1;10:1057. Originally published 2021 Oct 18. [Version 2] doi: 10.12688/f1000research.73161.2 (PMC10521057; doi:10.12688/f1000research.73161.2)
Supplement: Peer review history

# Reviewer report: Reviewer response for version 2

**DOI:** 10.5256/f1000research.138393.r154759

**Published:** 2022-11-04

**Authors:** Francisco Maria Calisto

**Affiliations:** 1 Institute for Systems and Robotics (ISR/IST), LARSyS, Instituto Superior Técnico (IST), University of Lisbon, Lisbon, Portugal; 2 Interactive Technologies Institute (ITI), LARSyS, Instituto Superior Técnico (IST), University of Lisbon, Lisbon, Portugal

The authors have covered all my concerns. The manuscript is now ready for indexing.

Is the work clearly and accurately presented and does it cite the current literature?

Partly

If applicable, is the statistical analysis and its interpretation appropriate?

Yes

Are all the source data underlying the results available to ensure full reproducibility?

Yes

Is the study design appropriate and is the work technically sound?

Yes

Are the conclusions drawn adequately supported by the results?

Yes

Are sufficient details of methods and analysis provided to allow replication by others?

Yes

Reviewer Expertise:

Human-Computer Interaction, Health Informatics, Artificial Intelligence

I confirm that I have read this submission and believe that I have an appropriate level of expertise to confirm that it is of an acceptable scientific standard.

# Reviewer report: Reviewer response for version 1

**DOI:** 10.5256/f1000research.76792.r142271

**Published:** 2022-07-19

**Authors:** Rajasvaran Logeswaran

**Affiliations:** 1 Faculty of Information Technology, City University, Petaling Jaya, Malaysia

**Summary**:

 The paper reports on novel work undertaken in automated grading of pancreatic cancer using pathology images and deep learning. The classification is based on the grade of the cancer, instead of just detecting the presence or type of cancer, and uses pathology images as opposed to the conventional radiology images prevalent in most prior research efforts. The main advantage of the proposed system is that it overcomes the very tedious and error prone conventional pancreatic cancer grading method that requires manual microscopic examination of the cancerous tissues after biopsy. The reduced misdiagnosis promotes providing correct treatment planning for patients. Although using only a limited size training set, the proposed system was tested using 14 different ImageNet pre-trained models and achieved high accuracies of up to 95.61% for two different pathological stains. 

**Comments**:

 The paper is well written and the flow is both logical and guides the reader through the work in an easy to understand manner. The technical content is good with appropriate use of vocabulary and terminology. The subject matter is of current interest and the publication is a worthwhile contribution to the body of knowledge.

**Recommendations for Improvement**:

 Some points of consideration for improvement of the paper are listed below:

1. *Abstract - Results: *It would be advisable to change "small sample set" to "small training set", to better represent the effectiveness even when training was limited. This is especially so as deep learning is employed for the grading system.
2. *Introduction: *Consider changing "paper" in "The absence of such AI work motivates this paper to" to "project" or "research".
3. *References: *Although the references are fairly recent, the paper would benefit by additionally citing the most recent relevant publications from the current year. It should also be checked if "et al." can be used in the References or the names of all authors should be listed in the References section.
4. *Methodology: *In terms of language, better to change "The methodology of this work was done at" to "The research work was undertaken at".
5. *Figure 1: *Do use consistent style of verbs in the flowchart, e.g. change "Fine tuning" to "Fine tune" and "Training" to "Train".
6. *Pathology image procurement: *Consider changing "which is" to "that are".
7. *Image dataset: *Consider adding a comma before "which" in "images which is".
8. *Image data augmentation and normalization:* It is stated that the images were rescaled from [0,255] to [0,1]. However, was this as a grayscale intensity image or for each of the RGB (or other colour space) components? Do clarify as there are no further images shown, making it difficult to infer.
9. Equation numbering could be added.
10. *Effect of data augmentation: *Citation 23 appears to be out of place. Consider changing "is doing very well on the training set" to "performed well on the training set". The citations at he end of the last sentence should be moved to before the full-stop.
11. *Table 5*: Consider simplifying the caption to "Model accuracy after 100 epochs" or removing "for". Similarly for the *Table 6 *caption.
12. *Figure 3*: The names of the dataset do not tally with the description given earlier in the text. For consistency, "Blue" and "Purple" should be changed to "MGG" and "H&E", respectively. The y-axis of the graph should be labelled ("Mean f1-score"). Also ensure consistency of spelling for "f1-score" in the caption and text. Similarly, ensure consistency of the spelling "Mixed" dataset (cf. "mixed").
13. *Comparison between pre-trained models: *Consider deleting ", which performs better than their ancestor."
14. *Comparison between the best and the worst performing model:* Consider changing "imbalance classes" to "imbalanced classes". Also, "recalling class with fewer data" to "recalling classes with less data".

**Data availability**:

 The authors have made their data and results available for other researchers. This is useful as it allows for verification of the findings, should the need be, and can propagates further research in this area by helping overcome the scarcity of labelled data. The code used is also provided to assist other researchers.

**Conclusion:**

 The paper presents useful findings that would be beneficial in quick and accurate pancreatic cancer grading for correct timely treatment planning. The finding may also open avenues of research and implementation in for other types of cancers and tissue analysis. This work is a useful contribution to the body of knowledge in this domain.

Is the work clearly and accurately presented and does it cite the current literature?

Yes

If applicable, is the statistical analysis and its interpretation appropriate?

Yes

Are all the source data underlying the results available to ensure full reproducibility?

Yes

Is the study design appropriate and is the work technically sound?

Yes

Are the conclusions drawn adequately supported by the results?

Yes

Are sufficient details of methods and analysis provided to allow replication by others?

Yes

Reviewer Expertise:

Medical image processing

I confirm that I have read this submission and believe that I have an appropriate level of expertise to confirm that it is of an acceptable scientific standard.

# Reviewer report: Reviewer response for version 1

**DOI:** 10.5256/f1000research.76792.r142618

**Published:** 2022-07-18

**Authors:** Francisco Maria Calisto

**Affiliations:** 1 Institute for Systems and Robotics (ISR/IST), LARSyS, Instituto Superior Técnico (IST), University of Lisbon, Lisbon, Portugal; 2 Interactive Technologies Institute (ITI), LARSyS, Instituto Superior Técnico (IST), University of Lisbon, Lisbon, Portugal

In this manuscript, the authors are presenting the development of several DL (deep learning) models through transfer-learning for classifying pancreatic cancer grade from pathology images. Specifically, the authors performed data augmentation across a dataset of images to counter the low number of images and improve the validation accuracies of all pre-trained models. Overall, this is an exciting line of work, but the current draft of this manuscript has some minor concerns that must be addressed first before publication. Contributions, if so, must be described clearly. Additionally, the findings are also important attributes of such significant research work. As follows, further comments are detailing improvements for the next iterations of the manuscript.

**Strengths**

**1.1.** The manuscript addresses key concerns about transfer-learning for classifying pancreatic cancer;

**1.2.** The manuscript brings forward novel perspectives for DL developments in a specific medical domain;

**Weaknesses**

**2.1.** Contributions and impact for this scientific community are not clear;

**2.2.** Findings and implications of the work are merely discussed;

**Requested Changes**

 There is much to appreciate in this manuscript, as it investigates several DL models for classifying pancreatic cancer. However, the provided findings and work implications are not enough, and the discussion section would need to be considerably strengthened by a more robust engagement with the cited literature. I would encourage the authors to think about how their findings extend or add to both DL and medical literatures in this space in order to refine their contribution to both fields. For instance, it would be interesting to understand the application of these DL models to a real scenario [1, 4]. At least, a small discussion on how could these techniques help real problems, such as different medical domains [2, 3, 5].

 The paper needs a brief proofreading. Be aware of tenses also. It would be wise to proofread the paper and correct these, as well as other spelling errors.

 The related work seems scarce for a work around the healthcare domain. The paper must discuss recent work in a methodological sense and base their work on the proposed approach. A lot of work is already available into this topic [1, 3, 5]. Specifically, the scientific community has numerous works on this topic [1, 5, 7], but authors could also map some literature around other domains [5, 6, 8], where these level of approaches are essential.

 To conclude, the paper is almost ready for indexing and the authors did a splendid work. However, I strongly recommend the authors improve the manuscript for the next iterations of the work, as this is a chief importance domain.

**Missing References**

 [1] https://doi.org/10.1259/bjr.20220072

 [2] https://doi.org/10.1016/j.artmed.2022.102285

 [3] https://doi.org/10.1007/s00521-022-07183-8

 [4] https://doi.org/10.1016/j.ijhcs.2021.102607

 [5] https://doi.org/10.1007/978-981-15-9735-0_2

 [6] https://doi.org/10.1145/3399715.3399744

 [7] https://doi.org/10.1371/journal.pone.0195621

 [8] https://doi.org/10.1145/3132272.3134111

Is the work clearly and accurately presented and does it cite the current literature?

Partly

If applicable, is the statistical analysis and its interpretation appropriate?

Yes

Are all the source data underlying the results available to ensure full reproducibility?

Yes

Is the study design appropriate and is the work technically sound?

Yes

Are the conclusions drawn adequately supported by the results?

Yes

Are sufficient details of methods and analysis provided to allow replication by others?

Yes

Reviewer Expertise:

Human-Computer Interaction, Health Informatics, Artificial Intelligence

I confirm that I have read this submission and believe that I have an appropriate level of expertise to confirm that it is of an acceptable scientific standard, however I have significant reservations, as outlined above.

## References

[1] : Pancreatic cancer, radiomics and artificial intelligence. *Br J Radiol* .2022; 10.1259/bjr.20220072 20220072 10.1259/bjr.20220072 35687700PMC10996946

[2] : BreastScreening-AI: Evaluating medical intelligent agents for human-AI interactions. *Artif Intell Med* .127: 10.1016/j.artmed.2022.102285 102285 10.1016/j.artmed.2022.102285 35430044

[3] : Detection of ring cell cancer in histopathological images with region of interest determined by SLIC superpixels method. *Neural Computing and Applications* .2022; 10.1007/s00521-022-07183-8 10.1007/s00521-022-07183-8

[4] : Introduction of human-centric AI assistant to aid radiologists for multimodal breast image classification. *International Journal of Human-Computer Studies* .2021;150: 10.1016/j.ijhcs.2021.102607 10.1016/j.ijhcs.2021.102607

[5] : Deep Learning-Based Medical Image Analysis Using Transfer Learning.932: 10.1007/978-981-15-9735-0_2 19-42 10.1007/978-981-15-9735-0_2

[6] : BreastScreening: On the Use of Multi-Modality in Medical Imaging Diagnosis. *AVI '20: Proceedings of the International Conference on Advanced Visual Interfaces* .2020; 10.1145/3399715.3399744 10.1145/3399715.3399744 Reference source

[7] : Identifying tumor in pancreatic neuroendocrine neoplasms from Ki67 images using transfer learning. *PLoS One* .2018;13(4) : 10.1371/journal.pone.0195621 e0195621 10.1371/journal.pone.0195621 29649302PMC5896941

[8] : Towards Touch-Based Medical Image Diagnosis Annotation. *ISS '17: Proceedings of the 2017 ACM International Conference on Interactive Surfaces and Spaces* .2020; 10.1145/3132272.3134111 10.1145/3132272.3134111 Reference source

# Response

**Published:** 2022-09-07

**Authors:** Wan Siti Halimatul Munirah Wan Ahmad

Thank you Prof. F.M.Calisto for the encouraging comments for improving our article.

 We have noted the following changes (new texts are emphasized in Bold):

Revised Introduction:

 Pancreatic cancer is one of the most lethal malignant neoplasms in the world,1 developed when cells multiply and grow out of control in the pancreas,2 forming cancer cells caused by cells mutation in their genes.3 Doctors commonly perform a biopsy to diagnose cancer when physical examination or imaging tests like magnetic resonance imaging (MRI) and computerized tomography (CT) scans are insufficient. In pancreatic cancer, grading is essential for planning treatment but is currently done using a meticulous microscopic examination.4 **Limited work found on analysis of pathological images for pancreatic cancer. Niazi et al. [Suggested Reference 7] presented a deep learning method to differentiate between pancreatic neuroendocrine tumor and non-tumor regions based on Ki67 stained biopsies. The purpose is for the quantification of positive tumor cells in a hotspot.** Up to now, there has been no successful implementation of artificial intelligence (AI) for classifying pancreatic cancer grade. The absence of such AI work motivates this paper to use transfer-learning to grade pathological pancreatic cancer images using 14 deep learning (DL) models. This work can facilitate an automated cancer grading system to address the exhaustive work of manual grading.

Revised Deep learning and related works section:

 Convolutional neural network (CNN) is a widely used deep learning (DL) algorithm in medical image-based classification and prediction.7 Several methods use CNN in cancer detection and diagnosis8 such as the Gleason grading of prostate cancer,9–11 colon cancer grading,12 breast cancer detection,13,14 and pancreatic cancer detection15–18 and classification.19 **AI has been proven to assist clinicians with better prediction and faster diagnosis for breast cancer screening [Suggested Reference 2].** However, the grading of pancreatic cancer with DL still needs comprehensive study.

New subsection at the end of Introduction:

**Contributions:**

**This work presents an automated grading system focusing on pancreatic cancer from pathology images, which has not been done before to the best of our knowledge. The work also contributes a comparison of performance for 14 DL models on two different pathological stains, namely the May-Grünwald-Giemsa and haematoxylin and eosin.**

Revised Effect of data augmentation section:

 This experiment was done with the first cross-validation set of the mixed dataset, to observe how data augmentation impacts a model training performance.23 Table 5 and Table 6 display the final accuracy and loss of training and validation set after 100 epochs. Without data augmentation in Table 5, it is evident that overfitting has occurred, because the model is doing very well on the training set but not on the validation set. With data augmentation, the validation accuracy improved, specifically on VGG19 model (54.83% to 77.22%). The training accuracies of other models are slightly reduced with data augmentation (except for VGG19) but it is normal as the model is learning newly transformed images. The validation loss also shows a reduction, as in Table 6, such as on NASNetLarge model from 3.36376 to 0.68587. Overall, these results show that data augmentation may reduce overfitting and improve model performance as reported in.10,13,14 **The reason behind this is the model is becoming more robust with data augmentation for getting to learn various transformed images of the limited size dataset. These kind of images are high-likely to exist in real-world applications, especially when it comes to unique human cells.**

Added last paragraph before Conclusion section:

**From the study, we can see that integrating AI into the diagnosis system can assist the pathologist in getting the suggestive grading based on the prediction. It is however meant to assist and not on decision-making. The future aim of this study is to have a platform for screening of pancreatic cancer biopsies.**

 We look forward to getting this article indexed. Thank you very much.
